# Dietary flavonoid intake in older adults: how many days of dietary assessment are required and what is the impact of seasonality?

**DOI:** 10.1186/s12937-017-0309-7

**Published:** 2018-01-12

**Authors:** Katherine Kent, Karen E. Charlton, Simone Lee, Jonathon Mond, Joanna Russell, Paul Mitchell, Victoria M. Flood

**Affiliations:** 1 Centre for Rural Health, Faculty of Health, University of Tasmania, Locked Bag 1322, Launceston, TAS 7250 Australia; 2 Faculty of Science, Medicine and Health, University of Wollongong, Wollongong, Australia; 3 Illawarra Health and Medical Research Institute, Wollongong, Australia; 4 Faculty of Social Sciences, University of Wollongong, Wollongong, Australia; 5 Centre for Vision Research, University of Sydney, Sydney, Australia; 6 Faculty of Health Sciences, University of Sydney, Sydney, Australia; 7 Western Sydney Local Health District, Sydney, Australia

**Keywords:** Flavonoids, Within-individual variation, Between-individual variation, Variance ratio, Dietary assessment, Seasonality

## Abstract

**Background:**

Within- and between-person variation in nutrient intake is well established, but little is known about variability in dietary flavonoid intake, including the effect of seasonality.

**Methods:**

Within- and between-individual variability of flavonoid intake, and intake of flavonoid subclasses was examined in older adults (*n* = 79; mean age 70.1 y (range: 60y-80y)), using three separate 4-day weighed food records (WFR) collected approximately 4 months apart. The effects of seasonality were also examined. Mixed-effects linear regression models were used to estimate within- and between-individual variance components for flavonoids and subclasses. The number of days of dietary assessment required for a high level of hypothetical accuracy was calculated from variance ratios.

**Results:**

Within- and between-individual variability was high for flavonoid intake, and intake of flavonoid subclasses, with variance ratios > 1. It was calculated that six days of WFR data are required for total flavonoid intake, and between 6 and 10 days was required for flavonoid subclasses. There was no effect of seasonality for total flavonoid intake or intake of flavonoid subclasses, with the exception that flavan-3-ol and flavanone intakes which were relatively low in summer, and in summer and winter, respectively.

**Conclusion:**

While the effects of seasonality on total flavonoid intake may be small, within- and between-individual variation associated with flavonoid intake assessment appears to be substantial across 12 days of WFR data in older adults. It is recommended that a minimum of 6 days of weighed food records are collected to minimise the impact of within- and between-individual variability on total flavonoid intake assessments in this population.

**Electronic supplementary material:**

The online version of this article (10.1186/s12937-017-0309-7) contains supplementary material, which is available to authorized users.

## Introduction

Flavonoids are a large group of naturally occurring plant-based compounds that are commonly consumed through a diet rich in fruit, vegetables, tea, wine and soy-based foods [1]. Habitual consumption of dietary flavonoids has been consistently linked with improvements in chronic conditions associated with ageing, certain cancers [2], cardiovascular [3] and neurodegenerative diseases [4–9]. Flavonoids are divided into six major classes: anthocyanins, flavan-3-ols, flavanones, flavones, flavonols and isoflavones [10].

Precise estimation of nutrient intake is essential for establishing a relationship between diet and disease. Flavonoids are abundant, wide-spanning and diverse in the human diet, and their quantity in foods is heavily influenced by a food’s growth and processing conditions [11]. For these reasons, estimations of dietary flavonoid intake need to take into account their complexity and variability. There are substantial variations in population estimates of dietary flavonoid intake [12–17], which may lead to inconsistent associations with health outcomes. A recent review reported a wide range for mean total flavonoid intakes of between 209 to 1017 mg/d (mean 435 mg/d) in Australian, European, and US adult populations [12]. This variability may relate to true differences in dietary patterns, such as differences in the food supply and cultural eating patterns between countries [18, 19]. However, it may also reflect well-known limitations associated with the assessment methods typically used to assess flavonoid and subclass intake [12], described below.

To determine the flavonoid composition of a diet, dietary intake data needs to be cross-referenced with a flavonoid-specific food composition database (FCDB). The dietary assessment method most commonly applied in the literature to determine flavonoid intakes is retrospective analysis of FFQs that aren’t developed or validated to measure flavonoid intakes specifically [20, 21]. This method, while useful when analysing large existing datasets has limitations for the accurate assessment of flavonoid intakes and can lead to inaccurate results, as often within the FFQs, foods which are nutritionally similar are grouped together (to make the FFQs shorter -i.e., green and red grapes), but these food items often possess very different flavonoid profiles. In addition to the well-known limitations of dietary assessment methods in general [22], in the case of flavonoids, there are additional methodological issues relating to the choice of FCDBs used to assign flavonoid content information to dietary data. These issues relate to the completeness and appropriateness of the flavonoid FCDB [12], which is in turn related to availability of analytical food data [23]. In Australia, for example, very little analytical food data exists for the flavonoid composition of foods, meaning that there are no Australian-specific flavonoid FCDBs to use. Additionally, flavonoid FCDBs are unable to account for inherent variability of the flavonoid composition of foods [11], which may fluctuate according to cultivar type, season, and/or processing and preparation methods [24]. Lastly, one recent study demonstrated significant variations in estimates of flavonoid intake when two different flavonoid FCDBs were applied to the same dietary data [25]. A comparison of the anthocyanin content of fruits and vegetables demonstrated marked variability in anthocyanin content values yielded by three different food composition database sources, namely the USDA tables, Phenol-Explorer and an Australian- specific flavonoid subclass (anthocyanin) database [23].

Differences in reported flavonoid intakes may be also be attributed to bias associated with different dietary assessment methodologies, leading to further errors in estimation of intake. For example, a Food Frequency Questionnaire (FFQ) and a 24 h diet recall would produce fundamentally different estimates of flavonoid intake, given the inherent differences in the recall and reporting periods of each tool. When assessing flavonoid intake, the majority of studies have applied a FFQ [26–28] to capture habitual intake, while fewer studies have utilised either single [29, 30] or multiple 24 h recalls [31], diet history methods [32] and food records [33]. The use of FFQs to determine flavonoid intake has limitations, as often a retrospective secondary analysis of flavonoid intake is conducted [34] from a FFQ tool that has not specifically been designed to assess flavonoid intake. Often, these tools group food items which are nutritionally similar but which possess very different flavonoid profiles. This is especially relevant for assessing fruits and vegetables [23]. Until recently [35–38], there has been a lack of validated dietary tools for estimating flavonoids and flavonoid subclasses, which is a major limitation to progress in establishment of dietary recommendations.

The known variability associated with estimating dietary flavonoid intake is often attributed to the aforementioned limitations of dietary assessment methodologies, with no consideration of the potential influence of within-individual variation (the inherent day-to-day fluctuation) in flavonoid intake. However, within-individual variation could be significantly contributing to the reported differences in population-based estimates of dietary flavonoid intake. There is substantial within- and between-individual variation for all dietary components, and it is generally well established that macronutrients show smaller variation than micro-nutrients [39]. Research has established that the number of days of dietary assessment required for accurate estimation of macronutrients intake is a 7-d recording period. However, the majority micronutrients require a longer time period (but less than 1 month) [40]. It has previously been hypothesised that ‘antioxidant’ dietary components would require more days of dietary assessment than macronutrients, but one study has shown that total flavonoid intakes would require 8 days of dietary assessment, but 10 days would be needed for energy assessment in the same population.

Despite this preliminary analysis, the number of days of dietary data needed to precisely assess flavonoid intakes is currently unclear, with only the one study addressing this issue to date in younger adults and only in relation to total flavonoid and isoflavone intake [40]. The inherent differences in eating patterns between younger and older adults, underpins the different major dietary sources of flavonoids in these groups, where the contributions of wine and tea to total flavonoid intake increases with age [41], and given these differences, a focused investigation on the variability in flavonoid intake for older adults is warranted. Information on within- and between-individual variation in flavonoid and subclass intake can be used to calculate the number of days of dietary assessment that are required to precisely estimate intakes of these food components.

Also of potential relevance when considering variability in flavonoid intake assessment, is the potential influence of seasonality. The influence of season on dietary consumption patterns has been established [42]. Seasonality has been shown to influence nutrient [43] and antioxidant [44] intakes, and may influence food availability [45]. However, the effect of seasonality on dietary flavonoid intake has not yet been adequately investigated. Given that fruits and vegetables are major sources of dietary flavonoids, the effect of seasonality on flavonoid intake could be significant.

The primary aims of the current research were: (1) to assess the between and within-individual variability of dietary flavonoid intake; and (2) to calculate the number of days required to assess usual intake of flavonoids and flavonoid subclasses within a defined level of accuracy using 12 days of weighed food record (WFR) data. A secondary aim of the research was to determine if seasonality impacted on total flavonoid or flavonoid subclass intake in this population.

## Methods

### Study population

The Blue Mountains Eye Study (BMES) is a longitudinal, population-based study of chronic health outcomes in residents aged 49 years and over in a defined area (the Blue Mountains, population of approximately 80,000 in 2016) west of Sydney, Australia [46]. All procedures of the Blue Mountains Eye Study were approved by the Human Research Ethics Committees of the University of Sydney and the Western Sydney Area Health Service, and were conducted adhering to the tenets of the Declaration of Helsinki. Written, informed consent was obtained from all study participants and de-identified data was provided to the research team. The dietary assessment methods utilised in this study have been described in detail elsewhere [46]. Briefly, twelve days of WFRs, comprising three separate 4-day WFRs were collected approximately 4 months apart in 1994 in a randomly selected sub-sample of the BMES cohort (*n* = 79). The WFR data were collected in the sub-sample for the purpose of validating a FFQ administered in the full population group [47], and the sample size was selected based on validation for energy intake (for full details please see [47]). The sub-sample comprised 45 females (57%) and 34 males with a mean age of 70.1 years (age range: 60y to 80y) and a mean body mass index (BMI, kg/m^2^) of 21.3 (± 3.3) [47].

### Flavonoid and subclass assessment

The 12 days of WFR data were selected to provide a comprehensive assessment of flavonoid intake, and given a previous study [40] showing that 8 days of dietary data are needed to calculate flavonoid intakes precisely, this data was likely to be robust enough for the proposed analysis. A strength of this dataset is that it is collected for 4-day periods over three separate time points, which allows for the analysis of within-individual variation in flavonoid intake. The dietary data were collected to reflect intake over a 12-month period, thereby spanning the different seasons [47–49]. WFR data was provided to the research team in a Microsoft Access (2010) database, which was developed for the purpose of storing and managing the large BMES data set. The dietary data comprising the WFRs were cross-referenced with the USDA Database for the Flavonoid Content of Selected Foods (Release 3.1) [24] to assign each food reported a total flavonoid value and a value for each flavonoid subclass: flavonols, flavan-3-ols, anthocyanins, flavones, flavanones. Isoflavone intake was not assessed as isoflavone consumption in Australia is very low (14) (isoflavones are largely provided by soy foods) and the isoflavone content of foods is not reported in the USDA flavonoid database. The content data was assigned based on the most similar and appropriate food/beverage available in the reference USDA flavonoid database. The USDA Database was chosen as our reference database as internationally it is one of the most comprehensive and commonly applied flavonoid FCDBs, despite containing limited information pertaining to some cooked foods. Additionally, the flavonoid content values may not have accurately reflected that of Australian-specific produce. However, the use of the USDA database to determine the flavonoid intakes is justified in this study given the lack of Australian-specific data for total flavonoid intakes [23].

After flavonoid-contents were assigned to the WFR data, the dietary data was linked with the population characteristics stored in the Excel Access (2010) database, using the query tool. The linked data table was then exported to SPSS version 23.0 (IBM Corporation, Somers, NY, USA) for statistical analysis. The mean and range of flavonoid intake per 4-day food record and intake of flavonoids and flavonoid subclasses per person per day have been reported previously, in addition to the major sources of flavonoids and flavonoid subclasses [17].

### Statistical analysis

Data analysis was performed using SPSS version 23.0 (IBM Corporation, Somers, NY, USA). Analysis indicated that the distributions of total flavonoid and all subclass intakes showed departure from normality.

### Calculation of number of days required for assessing usual nutrient intakes

Untransformed data for flavonoid and subclass intake were used in the analysis for within- and between-individual variation, in line with previous research [50, 51], for three reasons. First, the data (estimates of the relative contributions of variance for each dietary variable) were not substantially affected by log transformation. Second, transforming data would have introduced further error associated with transformation/back transformations, and previous research indicated that transformation did not improve the assumption of homoscedasticity across variables [52]. Third, untransformed data was presented in a meaningful unit (mg), and transforming data would have created difficulty with interpretation the results [53].

For total flavonoids and each subclass, median intake, mean intake, standard deviation (SD), and the within- and between-person variations were calculated using a mixed-effects regression model with a restricted likelihood estimator [40]. Mean within-person variation was determined and two coefficients of variation (CV) were calculated: CVw [(√within-person variation)/mean] × 100, and CVb [(√between-person variation)/mean] × 100. The within-to-between individual variance ratio (CVw^2^/CVb^2^) was then determined. The number of days (D) required for assessing usual intake of flavonoids or flavonoid subclasses uses a hypothetical correlation coefficient (*r*) between the observed and the true intakes, given by the formula proposed by Black et al. [39, 54]. As *r* increases, the proportion of individuals correctly classified increases [39]. For the current study, *r* ≥ 0.9 was selected in order to accurately classify 80% of individuals into thirds of a distribution with 90% confidence and ensure <1% of individuals are misclassified [40]. D is influenced by the variance ratio, whereby if the within-individual variance observed is smaller than the between-individual variation, a smaller number of repeated measures will be needed. D also depends on the selected *r*. Therefore, depending on hypothetical *r* selected, the resulting number of days of dietary assessment needed will increase or decrease, with *r* closer to 1 increasing the number of days required. To solve for D the following formula was applied: D = [(r^2^/1 − r^2^) × (CVw^2^/CVb^2^) [39].

### Assessment of seasonality

Each day of WFR data was categorized according to the season in which the data was collected, based on Australian conditions; summer: December – February; autumn: March – May; winter: June – August; spring: September – November. Both parametric (analysis of variance; ANOVA) and non-parametric (Kruskal-Wallis H) analyses were initially conducted to determine seasonality differences in estimated flavonoid intake (mg/day) and intake of flavonoid subclasses (mg/day), with α = 0.05. Since the results of these analyses were comparable, only results from the ANOVA are presented [55]. The results from the Kruskal Wallis test are available as supplementary material (Additional file 1).

## Results

### Days required for assessing usual flavonoid intakes

Table 1 presents the median and mean intakes, as well as presenting the standard deviation (SD) and interquartile range (IQR) to highlight the range of intakes for total flavonoids and each subclass. Coefficients of within- and between- individual variations for flavonoids and flavonoid subclasses are also presented. Overall, between-individual variation was greater than within-individual variation for total flavonoids and all subclasses, resulting in a variance ratio of more than 1. The number of days of dietary assessment required are presented in Table 1 and show that for total flavonoid intake 6 days of WFR data are required, 6 days are required for anthocyanins and flavan-3-ols, 8 days for flavones and flavanones and 10 days for flavonols (Table 1). Total flavonoid intake, anthocyanins and flavan-3-ols required less days of dietary assessment as they showed a smaller variance ratio, resulting from higher between-person variation than the other subclasses.

**Table 1:** Median, mean, SD and IQR for intake of flavonoid and flavonoid subclasses, coefficients of variation, within-to-between individual variance ratios and number of days to reach *r* ≥ 0.9 for 79 older adults

	Median	Mean	SD	IQR	CVw	CVb	Variance Ratio	D
	mg/day							
Flavonoid Total	581.84	678.69	498.53	619.58	136.18	116.74	1.36	6
Anthocyanins	1.05	6.73	12.70	7.88	80.40	72.78	1.22	6
Flavonols	24.06	28.04	33.29	21.21	139.70	91.79	2.32	10
Flavones	0.55	1.87	4.78	2.11	80.41	62.49	1.66	8
Flavan-3-ols	499.72	596.17	494.95	622.95	120.79	109.75	1.21	6
Flavanones	2.15	21.43	61.46	12.14	79.99	59.05	1.83	8

*SD* Standard Deviation, *IQR* Interquartile range, *CVw* [(√within-person variation)/mean] × 100, *CVb* [(√between-person variation)/mean] × 100, *Variance Ratio* (CVw^2^/CVb^2^), *D* days of dietary assessment needed, calculated by D = [(r^2^/1 − r^2^) × (CVw^2^/CVb^2^), *Mg* milligram

### Seasonal intake

Table 2 shows the seasonal differences in flavonoid intake and intake of flavonoid subclasses. When comparing total flavonoids intakes across seasons, there is > 60 mg difference between seasons, where intakes are highest in spring and lowest in autumn. However, there was no statistically significant difference detected between the seasons for total flavonoid intake using the ANOVA. Additionally, there was no statistically significant difference across seasons for flavonol or anthocyanin (highest in winter), or flavone (highest in autumn) subclasses. However, there were statistically significant differences across seasons for two flavonoid subclasses. For flavan-3-ol intake, a statistically significant difference between seasons was observed (*F*(3,944) = 2.79, *p* = 0.039). Post-hoc analysis using Tukey’s test indicated that flavan-3-ol intake was statistically significantly lower in summer than in spring (*p* = 0.029), with interpretation of the raw data showing a mean difference in intake of over 100 mg per day/person. A statistically significant difference between seasons was also detected for flavanone intake (*F*(3,944) = 3.85, *p* = 0.009). Post hoc analysis indicated that flavanone intake was higher in autumn than in summer (*p* = 0.017) and winter (*p* = 0.016), where flavanone intakes doubled in autumn, when compared to winter.

**Table 2:** Mean ± SD intake of flavonoids and subclasses (mg/day) according to season

		Flavonoid Total ^a^	Anthocyanin	Flavonols	Flavones	Flavan-3-ols*	Flavanones*
	n records	Mean ± SD					
Summer	177	652.35 ± 487.01	5.90 ± 13.17	26.01 ± 17.34	1.25 ± 2.85	501.93 ± 425.42*	15.26 ± 31.06*
Autumn	195	648.58 ± 400.21	5.95 ± 10.48	26.67 ± 18.32	2.29 ± 8.00	616.02 ± 488.97	34.06 ± 113.14
Winter	280	683.16 ± 548.65	7.78 ± 14.48	30.88 ± 40.94	1.92 ± 3.53	604.08 ± 526.50	17.06 ± 33.23*
Spring	296	710.06 ± 514.09	6.72 ± 11.90	27.48 ± 39.50	1.90 ± 3.77	631.97 ± 502.09	20.94 ± 44.08

All data are reported in milligrams (mg) of intake

*SD* standard deviation

*One way ANOVA *p* ≤ 0.05

^a^for flavonoid and subclass intakes in this population per person, per day, and according to gender please see [17]

## Discussion

This study shows, for the first time, that precise assessment of total flavonoid intake in older adults requires at least 6 days of weighed food records, and between 6 and 10 days to determine intake of specific flavonoid subclasses with an acceptable degree of accuracy. Season appears to influence intake of subclasses flavanones and flavan-3-ols, but not overall total flavonoid intake.

Substantial within-individual variation and between-individual variation was documented for both total flavonoid intake and intake of flavonoid subclasses in the current study. The within-individual variations ranged from around 80–140% and the between individual variation ranged from around 60–117%, which are both considerably greater than the range suggested for energy and macronutrients. Generally, the expected within- and between-individual variation for energy and other macronutrient intakes is around 25% in free-living subjects [56]. A number of studies have examined the between and within-individual variability of both macro and micro-nutrient and food intakes [57–60]. An early review by Bingham [61] identified the mean within-individual CV was lower for energy (23%), and macronutrients (carbohydrate (23%) and protein (27%)). The CV was reported to be greater for vitamins and minerals, such as calcium and iron (34%), ascorbic acid (63%) and retinol (131%). The review concluded that the wider the variation, the greater the number of days required for the reporting period [61]. It was suggested that 13 days of recording are necessary for 90% of the population to calculate mean energy intake with a standard error of ±10% [61]. Day-to-day variation in nutrient intake may be the result of an individual’s behaviour [62], such as differing meal patterns and food availability. For flavonoids, this variability may be attributed to the sporadic nature of consumption patterns of flavonoid-rich foods within the different flavonoid subclasses. For example, red wine or berries are major contributors to anthocyanin intake [63] but may not be consumed daily. The variation in flavonoid intakes between individuals is also high, with literature showing that sociocultural, economic and ecological factors may be responsible for the variation [64]. Additionally, small between-person variation may reflect a homogenous population, which does not appear to be the case in the this population, who varied in age and gender [47]. The within- and between-person variation for some dietary nutrients differs between genders, where women have shown higher CVs than males [40].

The major sources of variability when determining the flavonoid content of foods are well-known and include the cultivar, growing, processing, and preparation methods, and the variability associated with the analytical methods of flavonoid quantification [11]. Additionally, differences in a country’s food supply may limit the ability of an international flavonoid FCDB to accurately reflect the flavonoid composition of country-specific foods [65]. Studies frequently cite these factors as limitations in the interpretation of study findings. However, the potential impact of high within-individual variability on estimates of flavonoid intake has not been addressed. Whilst some studies have averaged repeated measures of flavonoid intake [66–68], so as to minimise the potential impact of within-individual variation, there is no description of the extent of the variability across different time points. The current findings suggest that studies collect as many days of dietary data as possible in order to minimize the effect of within-individual variability on estimates of flavonoid intake. However, increasing the number of days of dietary assessment to minimize this bias is associated with an increase in participant burden and may thereby detract from participant compliance with dietary recording. Therefore, statistically correcting for variability may be more appropriate for large epidemiological studies.

Several statistical methods exist to correct for within-individual variability in dietary intake data [69]. One method is to collect multiple days of 24-h recall data on each survey participant and average these data [69]. Another method is to apply a correction factor to the distribution. This method requires estimating the correction factor to be applied, by collecting multiple samples from a representative subset of the survey population for example [69]. This narrows the population distribution at the extreme ends due to accounting for within-individual variation. More sophisticated statistical modelling methods to account for variability include the Multiple Source Method (MSM) [70] and National Cancer Institute (NCI) [71] methods. However, these methods are usually applied to dietary information obtained by repeated short-term instruments, such as a repeated 24-h dietary recalls [70, 71] in large sample sizes.

Given that flavonoid intake is difficult to quantify, and in the absence of a gold standard approach, methods have been developed for application in various settings, including various techniques within the fields of dietary assessment and biomarker analyses [65]. A recent review [65] assessed the available tools to estimate dietary intake of polyphenols, including flavonoids, and identified little consistency across studies when applying FCDBs to estimate intake. Additionally, there is no consensus regarding which dietary assessment tool (e.g. FFQ, 24 h recall, food records etc.) should be utilized to provide the most valid measure of habitual flavonoid intake. However, the use of general FFQs not designed for the purpose of capturing flavonoid intake has been discouraged [12]. Recently, a flavonoid-specific FFQ for older adults was developed and validated [38]. Dietary flavonoid intake can also be determined by quantifying relevant biomarkers (e.g. intact phytochemicals or a related metabolite) found in various biological samples. However, there is currently no standardized protocol of how to perform these analyses or which biomarker to target [12]. Despite the significant problems associated with estimating flavonoid intake using a biomarker (such as within-individual variability in flavonoid metabolism [12]), future research should focus on the identification of appropriate and easily measurable biomarkers of flavonoid intake. This will be imperative in overcoming limitations associated with the estimation of flavonoid intake using dietary assessment.

There was no statistically significant effect of season on total flavonoid intake in the current study, despite flavonoid intake being relatively high in spring and relatively low in autumn. This finding is not aligned with findings from similar research, which showed that total antioxidant intakes in a Japanese population were highest in winter and lowest in summer [44]. The authors of this study were able to document differences in participants’ selection of food and beverages across the seasons, and therefore this analysis could be a consideration as a future extension of the current study. The analysis may be crucial to highlight if certain foods are responsible for contributing to the major differences flavanone and flavan-3-ol intakes across seasons. It is possible our lack of seasonal differences for total flavonoid intake reflects, in part, the way in which flavonoid values included in FCDBs are averaged across measurements when determining the flavonoid contents of foods, including different seasons [11, 24]. As flavonoid-specific FCDBs evolve, information on the influence of seasonality on the flavonoid content of foods may become more widely available. A limitation of this analysis is that the WFR data was collected across three seasons for each participant only. Ideally, dietary information would be collected mid-season, and in all seasons for each individual in future research.

The sample used for the current analysis was originally collected for a validation study of a FFQ developed for a prospective cohort study. The burden to participants entailed in the collection of twelve days of weighed food records is substantial and the sample size, while typical of validation studies of this nature, was relatively small. The reason for utilizing this dataset to estimate flavonoid intake in older adults relates to the richness of the dietary data. WFRs are likely to provide a more accurate estimation of flavonoid intake in comparison to other dietary assessment methods, such as repeated 24 h recalls. The dietary data collected in the total BMES sample was a FFQ, which grouped nutritionally similar foods (e.g., apples and pears). Given that such foods have significantly different flavonoid profiles, however, the FFQ may be unsuitable for accurately estimating flavonoid intake. Additionally, several major flavonoid contributing foods were not included in the BMES FFQ. Thus, despite the relatively small sample size, the depth of the dietary data from the WFRs in this group is a major strength when estimating flavonoid intake. We have previously compared flavonoid intake in this population to other national and international estimates for older adults, showing that older adults tend to consume higher amounts of dietary flavonoids when compared with younger age groups [17]. This may be related to higher intakes of tea and wine as people age [17]. It is difficult to compare flavonoid intakes in older adults across populations and studies because of differences in dietary assessment methods and the use of different FCDBs. Nevertheless, flavonoid intakes are reported to range from around 21.2 mg/day to 191.2 mg/day in this population [72].

An additional limitation of the current study is that the data used for this analysis was collected in the 1990s. However, in the BMES population, fruit and vegetable consumption did not significantly change from baseline to the 10-year follow up [73]. Some changes in dietary patterns related to fat (MUFA, PUFA, SFA) and total sugar (not CHO) intake [73] may have occurred during this period, but these macronutrients are not generally associated with flavonoid-rich foods. Nevertheless, the generalizability of the study findings may be limited by the changing food supply. Despite the age of the comprehensive dietary data used by this study. The USDA database chosen as our reference flavonoid FCDB was comprehensive enough to assign the WFR food items flavonoid content values. However, the validity of using a current (present-day) flavonoid FCDB to retrospectively assign flavonoid contents to foods collected approximately two decades earlier is uncertain, and these methodological limitations should be considered when interpreting the findings of this research. This study utilised an international FCDB, and therefore the potential inaccuracy of the flavonoid content of foods for Australian produce is a limitation of this study. The USDA [1] recognises that flavonoid contents in foods are influenced by cultivar types, and the growth and processing conditions of foods, but this is an issue across all flavonoid FCDBs. Therefore the USDA database was an appropriate choice for this study as it is a comprehensive resource and is commonly applied across studies. However, improvements in country-specific flavonoid FCDBs, ideally integrated into existing dietary analysis software, are vital to improve the accuracy and ease of flavonoid intake estimates in future studies.

Lastly, the sample size of the current study did not permit stratification of findings by gender, which is another limitation of the analysis. We have previously reported a significant difference in energy intake between men and women in the current study population [17] but a gender difference was not evident for flavonoid intake [17]. The vast majority of flavonoids are provided by tea, a low energy food, such that accounting for differences in energy intake is unlikely to uncover sex differences in flavonoid intake. Further research is needed to investigate the influence of energy intake, gender, or other confounders for diet, such as age and levels of physical activity on variations in flavonoid intake.

In conclusion, further research is needed to identify the determinants of the day-to-day variation in flavonoid and subclass intake within and between individuals, and whether a high variability in flavonoid intake has any biological implications in terms of metabolism, uptake and excretion [62]. Additionally, given the limitations of our study, further research is required to confirm our findings and to determine the appropriate number of days to accurately determine flavonoid intake. Comprehensive, Australian-specific flavonoid FCDBs are also needed, ensuring flavonoid content-values are representative across all seasons. Our study has shown that the within- and between-individual variation in flavonoid intake is considerable and needs to be accounted for in dietary assessment methodology. Additionally, the collection of dietary data in different seasons may not significantly influence estimates of total flavonoid intake but may influence the reported intakes for flavanones and flavan-3-ols. The findings of this study suggest that at least 6 days of weighed food records for total flavonoid intake, and up to 10 days for individual flavonoid subclasses, should be collected to reduce the bias associated with within-individual variations in intake.

## Additional file

Additional file 1: Results of the Kruskal-Wallis H Test for determining differences in variability of intakes by season. (DOCX 14 kb)

## Abbreviations

BMES: Blue mountains eye study
CV: Coefficient of variation
CVb: Between-individual variation
CVw: Within-individual variation
FCDB: food composition database
FFQ: Food frequency questionnaire
USDA: United States Department of Agriculture
WFR: Weighed food records
