# Supplementary material for: Dietary flavonoid intake in older adults: how many days of dietary assessment are required and what is the impact of seasonality?
Source: Nutr J. 2018 Jan 12;17:7. doi: 10.1186/s12937-017-0309-7 (PMC5767040; doi:10.1186/s12937-017-0309-7)
Supplement: Additional file 1: — Results of the Kruskal-Wallis H Test for determining differences in variability of intakes by season. (DOCX 14 kb) [file 12937_2017_309_MOESM1_ESM.docx]

**Additional file 1:** Results of the Kruskal-Wallis H Test for determining differences in variability of intakes by season.

|  |  |  |  | **mean rank score** | | | |
| --- | --- | --- | --- | --- | --- | --- | --- |
|  | **χ2** | **df** | **p value** | **summer** | **autumn** | **winter** | **spring** |
| **Flavonoid Total** | 1.89 | 3 | 0.596 | 458.07 | 474.61 | 467.43 | 490.93 |
| **Anthocyanin** | 3.785 | 3 | 0.286 | 449.51 | 458.84 | 489.35 | 485.71 |
| **Flavonols** | 4.11 | 3 | 0.25 | 467.70 | 473.65 | 500.21 | 454.81 |
| **Flavones*** | 9.41 | 3 | 0.024 | 423.76 | 472.36 | 484.34 | 496.94 |
| **Flavan-3-ols*** | 9.14 | 3 | 0.027 | 422.1 | 491.88 | 472.56 | 496.22 |
| **Flavanones*** | 10.39 | 3 | 0.015 | 442.66 | 524.35 | 457.09 | 477.17 |

*Flavone was determined as statistically significantly different using the Kruskal-Wallis H test, but post-hoc comparison did not determine any difference between seasons.
